# Decoding liver fibrogenesis with single-cell technologies

**DOI:** 10.1093/lifemedi/lnac040

**Published:** 2022-09-29

**Authors:** Tingting Zhou, Musunuru Kiran, Kathy O Lui, Qiurong Ding

**Affiliations:** CAS Key Laboratory of Nutrition, Metabolism and Food Safety, Shanghai Institute of Nutrition and Health, University of Chinese Academy of Sciences, Chinese Academy of Sciences, Shanghai 200031, China; Department of Medicine, and Department of Genetics, Cardiovascular Institute, Perelman School of Medicine at the University of Pennsylvania, Philadelphia, PA 19104, USA; Department of Chemical Pathology and Li Ka Shing Institute of Health Sciences, Prince of Wales Hospital, The Chinese University of Hong Kong, Shatin, Hong Kong SAR, China; CAS Key Laboratory of Nutrition, Metabolism and Food Safety, Shanghai Institute of Nutrition and Health, University of Chinese Academy of Sciences, Chinese Academy of Sciences, Shanghai 200031, China; Shanghai Jiao Tong University Affiliated Sixth People’s Hospital, Shanghai 200233, China; Institute for Stem Cell and Regeneration, Chinese Academy of Sciences, Beijing 100101, China

**Keywords:** fibrotic niche, non-parenchymal cells, zonation, stellakines, lipid-associated macrophage (LAM)

## Abstract

Liver fibrogenesis is a highly dynamic and complex process that drives the progression of chronic liver disease toward liver failure and end-stage liver diseases. Despite decades of intense studies, the cellular and molecular mechanisms underlying liver fibrogenesis remain elusive, and no approved therapies to treat liver fibrosis are currently available. The rapid development of single-cell RNA sequencing (scRNA-seq) technologies allows the characterization of cellular alterations under healthy and diseased conditions at an unprecedented resolution. In this Review, we discuss how the scRNA-seq studies are transforming our understanding of the regulatory mechanisms of liver fibrosis. We specifically emphasize discoveries on disease-relevant cell subpopulations, molecular events, and cell interactions on cell types including hepatocytes, liver sinusoidal endothelial cells, myofibroblasts, and macrophages. These discoveries have uncovered critical pathophysiological changes during liver fibrogenesis. Further efforts are urged to fully understand the functional contributions of these changes to liver fibrogenesis, and to translate the new knowledge into effective therapeutic approaches.

## Introduction

Liver disease has become a health burden in the world. Estimates suggested that major liver diseases cause over 2 million deaths per year, accounting for ~4% of all deaths worldwide [1]. In China, over one-fourth of the population were estimated to have liver diseases, and the incidence is still arising [2]. The major underlying pathology for liver failure and end-stage liver diseases are repetitive liver injuries, which ultimately result in liver fibrosis and even cirrhosis. However, despite that several classes of pharmacological agents have been developed to treat liver fibrosis, no approved therapies are currently available in clinic [3–6].

Liver fibrosis is characterized by excessive accumulation of extracellular matrix proteins (ECMs), which form a fibrous scar [7]. Previous studies using experimental rodent disease models and patient samples with liver fibrosis have unveiled key molecular mechanisms underlying liver fibrogenesis. The primary cells contributing to the excessive secretion of ECMs in the liver are the activated myofibroblasts [7]. However, the events leading to myofibroblast activation usually involve multiple cell types in liver tissue, including hepatocytes, immune cells, and endothelial cells, as well as the complex interplay between them. These cell types, together with intricately distributed ECMs and other environmental factors (e.g. pro-fibrotic factors), form a unique local microenvironment that promotes fibrosis, which is termed as liver fibrotic niche. Resolving the detailed composition of the fibrotic niche will certainly help to develop treatments for fibrosis.

The rapidly developing single-cell RNA sequencing (scRNA-seq) technology allows to capture of the heterogeneity of cell types and states at unprecedented resolution, enabling thorough characterization of cellular alterations under healthy and diseased conditions, thus unbiased exploration into tissue biology and disease mechanisms. In recent years, a number of liver scRNA-seq studies focusing on resolving the fibrotic niche under different disease conditions have been performed (Table 1). In this Review, we discuss how these scRNA-seq studies are transforming our understanding of the regulatory mechanisms of liver fibrosis. As Ramachandran *et al.* have nicely summarized the discoveries from liver scRNA-seq studies carried out by 2020 [26], the focus of this Review is more inclined to new discoveries generated from scRNA-seq studies in fibrotic livers in recent 2 years, with special emphasis on cell types including hepatocytes, liver sinusoidal endothelial cells (LSECd), myofibroblasts, and macrophages.

**Table 1.** Summary of single-cell RNA sequencing studies in liver fibrosis

References	Species	Platform(s)	Lineages represented	Highlights	Data availability	Animal model(s)
[8]	Mouse	10× Chromium; Smart-seq2	Mesenchyme	Characterization of the hepatic mesenchyme in healthy and fibrotic mouse liver; study of the functional zonation of HSC; identified LPAR1 as a therapeutic target to treat liver fibrosis	GEO: GSE137720 http://livermesenchyme.hendersonlab.mvm.ed.ac.uk	C57BL/6 mice with i.p injection of CCl4 twice-weekly for 6 weeks; or male Wistar-Han rats fed with CDHFD for 12 weeks
[9]	Mouse	10× Chromium	Mesenchyme	Interrogation of HSCs and myofibroblasts in healthy and fibrotic mouse liver	GEO: GSE132662	C57BL/6 mice with i.p injection of CCl4 three times per week for 3 weeks
[10]	Human, mouse	10× Chromium	Epithelia, immune, endothelia, and mesenchyme	Cell atlas of human healthy and fibrotic liver; defined scar-associated macrophages, and endothelial cells; interactome analysis between scar-associated populations within the fibrotic niche	GEO: GSE136103 http://www.livercellatlas.mvm.ed.ac.uk	Adult male C57BL/6JCrl mice with i.p CCl4 injection twice-weekly for 4 weeks
[11]	Human	10× Chromium	Epithelia, immune, endothelia, and mesenchyme	Analysis of lymphatic endothelial cells in healthy and chronically diseased human liver	GEO: GSE129933	N.A.
[12]	Mouse	10× Chromium	Epithelia, immune, endothelia, and mesenchyme	Cell atlas of healthy and NASH mouse liver; defined NASH-associated macrophages; study of HSCs as a hub of intrahepatic signaling via stellakines	GEO: GSE119340, GSE129516	C57BL/6J male animals under AMLN diet for20 weeks; or CDAHFD diet for 6 weeks
[13]	Mouse	10× Chromium	Immune (Myeloid leucocytes)	Characterization of myeloid cells in liver and bone marrow from healthy and NAFLD animals; defined “NAFLD myeloid phenotype” in liver myeloid cells and their bone marrow precursors during NAFLD progression	GEO: GSE131834	C57BL6/J mice fed with Western diet for 16 weeks
[14]	Mouse	10× Chromium	Immune	Cell heterogeneity within the macrophage pool in MAFLD; identified four subsets of hepatic macs in MAFLD, including ResKCs and 3 subsets of recruited macs, moKCs, pre-moKCs, and the distinct hepatic LAMs	GEO: GSE156059	C57BL6/J mice fed with Western diet and drinking water supplemented with fructose and sucrose for 12, 24, or 36 weeks
[15]	Mouse	10× Chromium	Immune, endothelia, and mesenchyme	Landscape study of cell- and disease-specific enhancers that identify key transcription factors driving myeloid cell diversity in NASH	GEO: GSE128338	C57BL/6J mice with a NASH-model diet for up to 30 weeks
[16]	Mouse	10× Chromium	Immune, endothelia, and mesenchyme	Analysis of sinusoidal cells from healthy and fibrotic mouse livers; Identified gene signatures in HSCs as markers of liver fibrosis	N.A.	Female C57BL6/J with i.p injection of CCl4 twice-weekly for 2–4 weeks
[17]	Mouse	Smart-seq2; 10× Chromium	Immune	Characterization of liver macrophages from healthy and HFD animals; identified two distinct Kupffer cell populations (KC1 and KC2); functional study of KC2 in diet-induced obesity	GEO: 168989	C57BL/6 mice fed with HFD for 9 or 18 weeks
[18]	Mouse	10× Chromium	Immune	Compositional study of the resident and recruited macrophages in NASH	GEO: GSE162651	C57BL6/J mice fed with HFD for 16 weeks
[19]	Human, mouse	10× Chromium; CITE-seq	Epithelia, immune, endothelia, and mesenchyme	Spatial proteogenomic single-cell atlas of healthy and obese murine and human liver; characterization of lipid-associated macrophages; evolutionary conserved BMP9/10-ALK1 axis essential for Kupffer cell development	GEO: GSE192742	C57BL/6J mice fed with Western diet for 24 or 36 weeks
[20]	Mouse	Drop-seq	Epithelia (hepatocytes)	Characterization of hepatocytes in responses to HFD; captured zonal differences of hepatocytes	GEO: GSE157281	C57BL6/J male mice fed with HFD for 12 weeks
[21]	Mouse	10× Chromium	Immune, endothelia, and mesenchyme	Cell atlas of fibrotic mouse liver from animals with or without myeloid depletion of YAP1; Functional study of myeloid YAP1 in liver fibrogenesis	GEO: GSE183018	Mice were i.p injected by CCl4 once every three days for 7 injections
[22]	Mouse	snRNA-seq2; Smart-seq2	Epithelia, immune, endothelia, and mesenchyme	Nuclear transcriptome analysis from frozen healthy and fibrotic liver tissue; revealed active crosstalk between gene dosage and spatial distribution of hepatocytes	ArrayExpress: E-MTAB-9333, E-MTAB-10223	C57BL/6 mice with i.p injection of CCl4 twice-weekly for 6 weeks
[23]	Mouse	BD Rhapsody	Epithelia, immune, endothelia, and mesenchyme	Cell atlas of healthy and diseased mouse liver with chronic injury	NA	C57BL/6 mice fed with CDE or DDC diet for 3 weeks
[24]	Human, mouse	N.A.	Epithelia, immune, endothelia, and mesenchyme	Integrative analysis of scRNA-seq data from chronic liver diseases; identified natural killer cells as a type of HCC-associated cell	GEO: GSE136103, GSE149614, GSE112271	N.A.
[25]	Human, mouse	SEAM; Geo-seq	Epithelia, immune, endothelia, and mesenchyme	Characterization of the spatial metabolic profile in human fibrotic liver; discovered special metabolic features in subpopulations of hepatocytes associated with fibrosis	https://doi.org/10.5281/zenodo.3951613	N.A.

CDAHFD, choline-deficient, amino acid-defined high-fat diet; CDE, choline-deficient, ethionine-supplemented; DDC, 3,5-diethoxycarbonyl 1,4-dihydrocollidinen; MAFLD, metabolic associated fatty liver disease; moKC, monocyte-derived macrophage; NAFLD, nonalcoholic fatty liver disease; NASH, nonalcoholic steatohepatitis.

## Hepatocytes

Hepatocytes make up 70%–85% of the liver mass, which are the major cell type in realizing multiple liver functions including protein synthesis, detoxification, glucose, and lipid metabolism. In response to injury, hepatocytes display altered gene expression and secretome profile, with several fibrogenic factors newly synthesized, including Notch, osteopontin, NADPH oxidase 4, TAZ, Indian Hedgehog, and TGFβ [27–30]. Recent studies, especially the studies using scRNA-seq analysis in mouse and human liver samples, all pointed out a general phenomenon that hepatocytes in the mammalian liver are not a homogenous population [31–33]. The concept of “hepatocyte zonation” has therefore been broadly accepted. Each hepatocyte is surrounded by a highly variable microenvironment, which is mainly created by the blood flow along the liver lobule radial axis, and the liver morphogenetic fields. This spatial variability results in zonal differences in hepatocyte functions, e.g. in metabolism and xenobiotic processing abilities. The characterization of hepatocyte zonation has been thoroughly reviewed [34].

In line with this, histological studies revealed distinct sensitivities of hepatocytes in specific regions to fat accumulation [35–37] or injury [31, 38–41]. For example, pericentral hepatocytes accumulate more lipids, e.g. arachidonic acid, which may account for increased oxidative damage observed in liver pericentral regions in nonalcoholic steatohepatitis (NASH) [36]. In a different aspect, specific hepatocyte subpopulations have been identified to possess enhanced proliferative capability in response to liver injury or in homeostatic renewal. These subpopulations of hepatocytes were found in the periportal region, the pericentral region, or stochastically distributed across the liver lobule. A subset population of periportal hepatocytes was identified to be the primary hepatocytes responsible for cell replenishment following chronic liver injury [38]. These hepatocytes express *Sox9* and other bile-duct-enriched genes at a low level, demonstrating a hybrid phenotype of both hepatocytes and biliary epithelial cells, and thus are termed hybrid hepatocytes [38], suggesting a progenitor-like status. A different study identified a subset of diploid cells positive of the Wnt-responsive gene Axin2 and early liver progenitor marker Tbx3. These diploid cells were found adjacent to the central vein, and were able to replace all hepatocytes along the liver lobule during homeostatic renewal [41]. Several other hepatocyte subpopulations were also identified with higher hepatic regenerative capacity. These subsets of hepatocytes either express high levels of telomerase [39], or are AFP-positive [31, 40]. Elegant proliferating tracing strategies and experiments with 14 murine fate-mapping strains also revealed regional hepatocyte generation in liver homeostasis and repair [42, 43]. These findings suggest that specific populations of hepatocytes may contribute more to liver cell replenishment under different physiological or pathological conditions. However, several other studies demonstrated limited contributions of Axin2 positive hepatocytes to liver homeostasis and regeneration, and pointed out that no special subset of hepatocytes has higher homeostatic and reparative potential, irrespective of their lobular location or ploidy status [44–46]. These studies argue against the zonal differences of hepatocytes in their regenerative capabilities under homeostatic or injury conditions, putting this as a remaining issue in the field [47, 48]. Nonetheless, under repetitive extraneous injuries, the liver gradually loses its regenerative ability [49–51], which contributes significantly to liver fibrosis. Further analysis combining lineage tracing and single-cell technologies may facilitate the study of cell heterogeneity in proliferative ability in response to liver damages, which will certainly be helpful in developing strategies to revive liver regeneration under diseased conditions.

**Figure 1.**
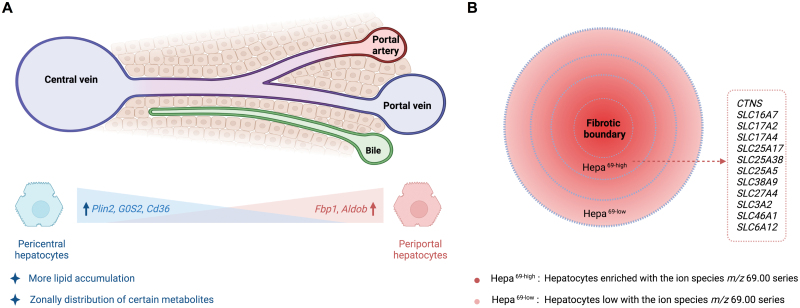
ScRNA-seq analysis of hepatocytes in fibrotic livers. (A) In mice under HFD, pericentral hepatocytes show upregulated genes in lipid formation (e.g. *Plin2, G0S2, and Cd36*), and accumulate more lipids; whereas periportal hepatocytes have increased expression of genes in glucose metabolism (e.g. *Fbp1*, *Aldob*). SEAM analyses with livers from wild-type mice show only zonal-distributed metabolites in pericentral regions. (B) SEAM analyses in human fibrotic liver tissues identify hepatocytes with specific metabolic features associated with their proximity to the fibrotic niche. In addition, fibrosis-proximal hepatocytes are enriched with expression of solute carrier transporter families. Hepa^69-high^, Hepa^69-low^: Hepatocytes with high or low level of ion species *m/z* 69.00 series in SEAM analysis.

ScRNA-seq analysis of hepatocytes in livers with chronic disease also revealed zonal differences in changes of gene expression [20]. Under high-fat diet (HFD) for 12 weeks, periportal hepatocytes showed upregulation of a small number of genes in glucose metabolism, e.g. *Fbp1*, *Aldob*; whereas pericentral hepatocytes displayed increased expression of genes in lipid formation, e.g. *Plin2, G0S2, Cd36*, which is consistent with the discovery that pericentral hepatocytes are prone to accumulate more lipids [35–37] (Fig. 1A). Another study explored the spatial metabolic profile at the single-cell level in liver tissue by applying the cutting-edge SEAM (spatial single nuclear metabolomics) method [25]. Interestingly, the application of SEAM to healthy mouse liver revealed zonal metabolic patterns in the central vein regions, but not in the portal node regions. In addition, with SEAM analysis in human fibrotic liver tissues, a subset of hepatocytes was discovered with specific metabolic features associated with their proximity to the fibrotic niche. These hepatocyte subpopulations with different metabolic features also had distinct transcription profiles, suggesting that the fibrogenic microenvironment could influence both cellular metabolic homeostasis and gene expression (Fig. 1B). In general, these studies have all revealed zonal differences in hepatocytes in response to liver injury or diet intervention, or in fibrotic condition. However, the major driven factors for these zonal differences, and how the hepatocyte zonation contributes to fibrogenesis are so far not clear.

## Liver endothelial cells

LSECs are highly specialized endothelial cells, which contain fenestrae and represent a permeable barrier between blood cells on one side and hepatocytes and hepatic stellate cells (HSCs) on the other side. The physiological function of LSECs has been described in detail in these reviews [52, 53]. In particular, LSECs possess the highest endocytosis capacity of human cells, and are critical in maintaining the quiescence of HSCs under physiological conditions. Whereas in fibrotic livers, LSECs undergo capillarization [53, 54], leading to HSC activation and fibrogenesis [52, 55, 56]. Importantly, different signals from LSECs may actually modulate liver regeneration and fibrosis in response to liver injury via pro-regenerative CXCR7-Id1 or pro-fibrotic FGFR1-CXCR4 angiocrine pathways, highlighting the crucial function of LSECs in liver fibrogenesis [55].

**Figure 2.**
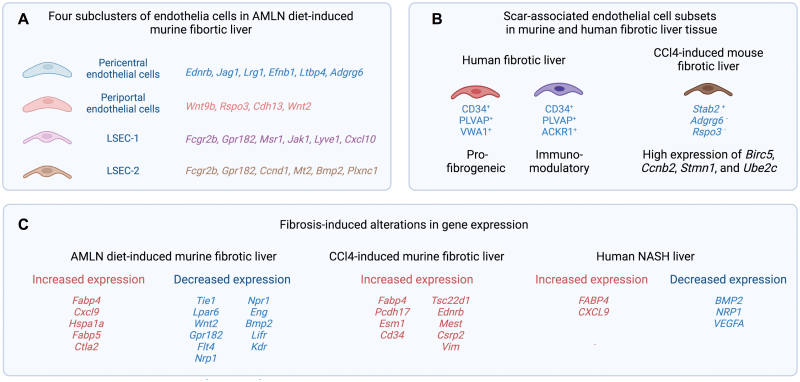
ScRNA-seq analysis of liver endothelial cells in fibrotic livers. (A) In mice under fibrotic induction by AMLN diet, four subclusters including pericentral and periportal endothelial cells, and two different subsets of LSECs are identified. (B) Discovery of two scar-associated endothelial cells in human fibrotic liver, and one in CCl4-induced murine fibrotic liver. (C) Endothelial cells from fibrotic livers display increased expression of genes in lipid metabolism and chemokine release, and decreased expression of genes in vascular development and homeostasis, in both humans and mice.

The scRNA-seq studies allow further investigations of the changes in specific status or subtypes of LSECs between healthy and fibrotic livers. Indeed, Ramachandran *et al.* identified scar-associated endothelial cell subpopulations (CD34^+^PLVAP^+^VWA1^+^ and CD34^+^PLVAP^+^ACKR1^+^) specifically in the fibrotic niche from human cirrhotic liver tissues [26]. The CD34^+^PLVAP^+^VWA1^+^ subcluster expresses clear pro-fibrogenic gene signatures with high levels of *PDGFD, PDGFB, LOX,* and *LOXL2*; and the CD34^+^PLVAP^+^ACKR1^+^ subcluster display an immunomodulatory phenotype, which may potentially function in leucocyte recruitment via ACKR1 (Fig. 2B).

Another study used murine NASH model induced by feeding with amylin diet (AMLN diet) for 20 weeks, in which clear liver fibrosis was induced [12]. In this study, four different groups of endothelial cells were identified, including periportal (high *Wnt9b*, *Rspo3*, *Cdh13,* and *Wnt2* expression) and pericentral (high *Ednrb*, *Jag1*, *Lrg1*, *Efnb1*, *Ltbp4*, and *Adgrg6* expression) endothelial cells, and two clusters of LSECs (high *Fcgr2b* and *Gpr182* expression), supporting the functional zonation of liver endothelial cells [33] (Fig. 2A). Although the transcriptomes are overall similar between the two different LSEC clusters, one LSEC cluster specifically expresses higher *Msr1*, *Jak1, Lyve1,* and *Cxcl10*, whereas the other has higher levels of *Ccnd1*, *Mt2*, *Bmp2*, and *Plxnc1*. The functional implications of these cluster-specific genes in LSECs are not clear [12]. Both scRNA-seq and pooled RNA-seq analyses on liver endothelial cells confirmed NASH-induced alterations of gene expression in all four endothelial subclusters [12]. Specifically, endothelial cells from NASH livers displayed increased expression of genes in lipid metabolism, antigen presentation, and chemokine release; whereas genes in vascular development and homeostasis were downregulated in NASH endothelial cells (Fig. 2C). In line with this, lipid accumulation was strongly increased in LSECs obtained from NASH animals. Further analysis with microarray dataset containing samples from healthy, nonalcoholic fatty liver disease (NAFLD) and NASH human patients confirmed similar observations as seen in murine NASH tissues. Compared to healthy human livers, expression levels of *CXCL9* and *FABP4* are upregulated in human NASH livers, and *BMP2*, *NRP1*, and *VEGFA* are downregulated in livers from both NAFLD and NASH patients (Fig. 2C). These results altogether demonstrate disrupted vascular and angiocrine signaling during NASH progression in both humans and mice [41].

A different study with fibrotic animal models induced by CCl4 treatment also reveal considerable gene expression changes in stabilin 2 (*Stab2*)-expression LSECs after fibrotic induction [16]. Genes otherwise expressed in nonsinusoidal liver endothelial cells are induced in these *Stab2*-expression LSECs, suggesting the activation and dedifferentiation of LSECs [16]. In addition, CCl4 treatment also results in enhanced expression of TGFβ-responsive genes in LSECs, including *Csrp2*, *Vim*, *Tsc22d1*, *Ednrb,* and *Mest* (Fig. 2C). Interestingly, a subcluster of liver endothelial cells, positive for *Stab2* but negative for *Adgrg6 or Rspo3* expression, was identified largely from CCl4-treated animals, with high expression of *Birc5, Ccnb2*, *Stmn1,* and *Ube2c*, indicating potential injury-associated LSEC proliferation (Fig. 2B). These studies altogether highlight the utility of scRNA-seq in defining disease-specific responses of a certain cell type with high resolution. More scRNA-seq studies focusing on LSECs and further comparative analysis between different studies may help identify common alterations of LSECs in liver fibrosis induced by different protocols, or conserved in mice and humans.

## Myofibroblasts

Myofibroblasts are considered the primary source of ECMs in the fibrotic liver which are not present in the healthy liver [7, 57, 58]. The major source of fibrogenic myofibroblasts are liver-resident activated HSCs and activated portal fibroblasts. Animal studies show that HSCs are primarily activated in livers under toxic injuries affecting the centrilobular and perisinusoidal regions; whereas both HSCs and portal fibroblasts are activated in cholestatic liver fibrosis caused by periportal injury [59]. In addition, human scRNA-seq data demonstrate that HSCs are likely the major source of collagen-producing myofibroblasts in the human fibrotic liver (Hijmans *et al*.). Bone marrow-derived fibrocytes, bone marrow-derived mesenchymal stem cells, and the epithelial-to-mesenchymal transition may also contribute to a small population of fibrogenic myofibroblasts, which are thoroughly reviewed elsewhere [4].

**Figure 3.**
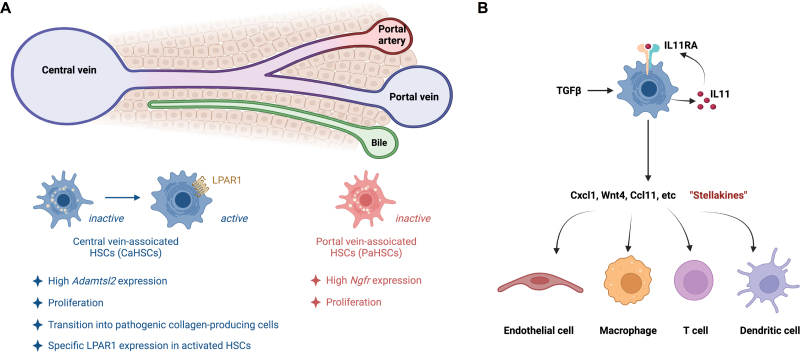
ScRNA-seq analysis of HSCs in fibrotic livers. (A) HSCs are classified by PaHSCs and (CaHSCs in both humans and mice, in which the CaHSCs present the dominant source of pathogenic collagen-producing cells following centrilobular liver injuries (e.g by CCl4 treatment). The *Lpar1* gene, encoding for the LPAR1, is identified with specific expression in collagen-producing HSC subsets, presenting a potential target to activated HSCs. (B) The study with murine NASH model induced by AMLN diet presents a unique function of HSCs as a hub in responding and organizing intrahepatic signals during liver fibrogenesis via secreting “stellakines.” The HSCs also create an autocrine IL11 signaling that stimulates stellakine secretion during fibrogenesis.

The functional zonation of HSCs has also been observed in both humans and mice, by which HSCs are classified by portal vein-associated HSCs (PaHSCs) and central vein-associated HSCs (CaHSCs) [8] (Fig. 3A). Markers genes conserved between the human and mouse are also identified, with the PaHSCs expressing high *Ngfr*, whereas the CaHSCs expressing high *Adamtsl2* [8]. In addition, the CaHSCs present the dominant source of pathogenic collagen-producing cells following centrilobular liver injury induced by CCl4 treatment [8]. Acute CCl4 injury results in similar levels of proliferation between CaHSCs and PaHSCs. However, only CaHSCs exhibit clear transitioning into pathogenic collagen-producing cells, which express high *Col1a1, Col3a1,* and *Acta2*, after acute CCl4 treatment [8]. Different sets of regulons are identified that may account for the transcriptional regulation of CaHSC activation following acute or chronic CCl4-induced liver injury, of which some are shared between both [8]. The common ones include *Egr2*, *Sox4*, *Plagl1*, *Rxra*, *Foxf1*, and *Klf7*, although additional functional analyses of these transcription factors in HSC activation are warranted. Remarkably, the *Lpar1* gene, encoding for the lysophosphatidic acid receptor 1 (LPAR1), is identified with specific expression in collagen-producing HSC subsets, in both mouse and human fibrotic livers. In line with this discovery, LPAR1 antagonism significantly reduces liver fibrosis in a murine NASH model induced by choline-deficient high-fat diet (CDHFD) *in vivo*, and suppresses human HSC contractility and activation *in vitro* [8], highlighting a potential strategy to target-activated HSCs.

Another interesting study using the murine NASH model induced by AMLN diet presents a unique function of HSCs as a hub in responding to and organizing intrahepatic signals during liver fibrogenesis [12] (Fig. 3B). HSCs secrete a panel of factors, termed “stellakines,” which are increased during NASH progression. Cellular interactome analysis indicates that these stellakines can act on liver endothelial and immune cells. In addition, HSCs express both *Il11ra1*, encoding a receptor of the interleukin (IL)-6 family, and its ligand *Il11*, creating an autocrine IL11 signaling that stimulates stellakine secretion. Moreover, the *Il11* mRNA expression is robustly induced in diet-induced NASH livers, pointing to a potential function of the autocrine IL11 signaling in augmenting stellakine secretion during NASH progression. In addition, a number of HSC-enriched receptors have also been implicated in liver fibrosis. These receptors include *Pdgfrb*, *Fgfr2, Ddr2*, *Ryk*, and *Lrp1*, many of which show altered expression in NASH livers, suggesting their functions in accepting signals and modulating cell fate switch of HSCs during NASH progression.

A different study on CCl4-induced mouse fibrosis model identified plasmalemma vesicle-associated protein (PLVAP) as a protein highly expressed in mouse and human HSCs [16]. PLVAP was previously shown to have a prominent role in maintaining vascular integrity and permeability [60, 61]. Interestingly, PLVAP expression is lost in activated HSCs upon injury, indicating a previously unknown function of HSCs in regulating sinusoidal remodeling during liver fibrogenesis [16]. Taken together, single-cell profiling of HSCs has greatly extended our understanding of this cell type in liver fibrogenesis. In addition to producing ECMs, HSCs are in the center of liver cell community that regulate liver homeostasis and disease progression. Specific targets identified in collagen-producing HSCs will allow the development of new strategies for fibrosis treatment.

## Inflammatory cells

The liver has a unique capacity in organizing effective immune responses against hepatotropic pathogens, which makes it a highly immunological organ, important in maintaining the local and systematic immune homeostasis [62, 63]. The utility of scRNA-seq technologies to analyze the liver immune system in healthy and diseased conditions has revolutionized our understanding of the diverse and complex immune regulations. Multiple studies highlight specific populations of immune cells that mediate particular functions in liver fibrogenesis. Macrophages are the most studied immune cells in the pathogenesis of liver fibrosis, we thus decide to specifically focus on new discoveries in liver macrophages here.

## Macrophages

Liver macrophages are unique in that they form the first line of defense against hepatotropic pathogens from the gastrointestinal tract, which contribute significantly to both liver fibrosis progression and resolution [64]. Liver macrophages consist of tissue-resident Kupffer cells (resKCs) derived from embryonic precursors from the yolk sac or the fetal liver [65, 66], and recruited monocyte-derived macrophages (MDMs) from the systemic circulation derived from bone marrow [67, 68]. The MDMs are considered to be the major source of TGFβ, which is a key mediator of liver fibrosis [69]. Under inflammation conditions during liver fibrogenesis, MDMs enter and expand the pool of liver macrophages [57]. Investigations into liver macrophages with single-cell technologies have unraveled specific dynamics and subsets of liver macrophages in liver fibrosis with great detail.

### Tissue-resKCs

**Figure 4.**
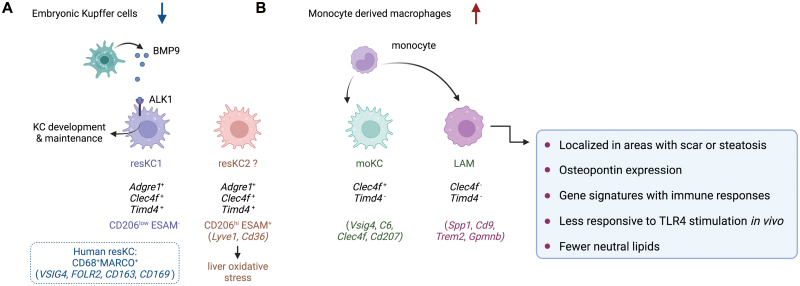
ScRNA-seq analysis of liver macrophages in fibrotic livers. (A) In fibrotic livers, the self-renew and survival of resKCs are severely impaired. In mice, two distinct resKCs are identified, with a major population as CD206^low^ ESAM^−^ and a minor population of CD206^hi^ESAM^+^. The latter may contribute to liver oxidative stress associated with obesity. However, the markers CD206 and ESAM of the minor subset are also expressed by LSECs, thus questioning that the CD206+ESAM+ population may actually reflect KC-LSEC doublets (shown by the question mark). The development and maintenance of KCs are regulated by the ALK1-BMP9/10 ligand-receptor pair between KCs and stellate cells. The CD68^+^MARCO^+^ macrophages in healthy human livers are defined as similar to Kupffer cells in mice. (B) MDMs have increased infiltration in fibrotic livers. Some of the newly recruited MDMs acquire similar phenotypes to resKCs, which are designated as moKCs. Besides moKCs, monocytes recruited to the liver also differentiate into a distinct subset of macrophages expressing high *Cd9*, *Trem2*, and *Gpmnb*, termed LAMs. The current known features of LAMs are summarized in the blue box.

In mice, resKCs are identified with high levels of *Adgre1*(F4/80), *Clec4f*, *Timd4,* and low level of *Itgam* (CD11b) (Fig. 4A). Interestingly, studies identified two functional distinct resKCs in mice, with a major population as CD206^low^ ESAM^−^ and a minor population of CD206^hi^ESAM^+^ [17, 70, 71]. Functional analyses of the CD206^hi^ESAM^+^ population suggest a significant contribution of this subset to liver oxidative stress associated with obesity [17]. However, the markers for the minor KC subset, namely CD206 and ESAM, are also expressed by LSECs, thus questioning that the CD206+ESAM+ population may actually reflect KC-LSEC doublets [19, 72, 73]. In humans, no validated markers to data of bona fide resKCs have been described. One study identified the CD68^+^MARCO^+^ macrophages in healthy human livers as similar to Kupffer cells in mice [40]. The other study using proteogenomic approaches suggested VSIG4 as the best human KC protein marker, with also FOLR2, CD163, and CD169 as useful markers to define human KCs [19]. While the murine KCs preferentially locate in periportal and mid zones [74, 75], human KCs are mainly mid-zonal located [19].

During the progression of NASH, the self-renew and survival of Kupffer cells are severely impaired, which are then replaced by MDMs for the maintenance of the liver macrophage pool [10, 14, 15] (Fig. 4). Importantly, compared to resKCs, replenished MDMs exhibit a more immature and pro-inflammatory status with limited ability for triglyceride storage [69, 76, 77]. The reduction in resKCs has not been observed during the early stages of steatosis, suggesting the high relevance of this event with liver fibrogenesis [78]. An evolutionarily conserved ALK1-BMP9/10 ligand-receptor pair between KCs (ALK1; encoded by *Acvrl1*) and stellate cells (BMP9/10 encoded by *Gdf1/Bmp10* respectively) has been demonstrated to be crucial in regulating both the development and maintenance of KCs in liver [19].In addition, studies focusing on changes in epigenetic landscapes in liver macrophages identify the function of transcription factor LXR during NASH progression, which may contribute to impaired Kupffer cell self-renew and survival [15]. Further studies on signals that are responsible for the epigenetic reprogramming and other regulators in this process may help to identify targets to prevent the loss of resKCs during NASH progression.

### MDMs

Under inflammatory conditions or when native resKCs are depleted, MDMs can replace dying embryonic resKCs [68, 79] (Fig. 4B). As also stated above, the liver macrophage population undergoes a marked expansion during NASH progression, in which the number of resKCs decreases, whereas infiltration of monocytes increases. In mice, some of the newly recruited MDMs acquire similar phenotypes to resKCs, which are designated as monocyte-derived macrophages (moKCs) [68, 79]. MoKCs express *Clec4f* and many other signature genes of KCs, but lack the expression of some typical ResKC genes such as *Timd4* [14].

Besides MoKCs, monocytes recruited to the liver also differentiate into a distinct subset of osteopontin-expression CLEC4F^−^ macrophages in mice [14]. These macrophages express high *Cd9*, *Trem2*, and *Gpmnb*, marker genes of lipid-associated macrophages (LAMs), which were first identified in obese adipose tissues from both mice and humans [80]. This subset of liver macrophages was thus termed LAMs as well [14, 19]. Compared to ResKCs and moKCs, mouse hepatic LAMs show gene signatures with biological processes that are more broadly associated with immune responses [14, 19]. However, LAMs are less responsive to *in vivo* TLR4 stimulation as compared to KCs [19]. Despite the high expression of genes associated with lipid metabolism in adipose LAMs [80], liver LAMs have a lower or equal expression of most lipid metabolism genes compared with the KC populations, except for *Lpl* and *Pparg*, and contain fewer neutral lipids than KCs [14].

One study identified similar TREM2^+^CD9^+^ scar-associated macrophages specifically expanded in fibrotic human livers and localized in areas of scarring [10]. However, the other study identified LAMs in all patients irrelevant to the status of liver fibrosis, only with a trend toward increased LAM proportions in livers with >10% steatosis [19]. In addition, liver LAMs show altered localization in both human steatotic livers and murine NAFLD models [19]. Specifically, LAMs localize in portal zones of nonsteatotic human and mouse livers, whereas primarily peri-centrally in zones with steatosis. The alteration of LAM localization suggests that MDMs are recruited to different locations in healthy and steatosis livers, where they differentiate into LAMs. Although subpopulations of fibroblasts with expression of *Ccl2, Cd44,* and *Vcam1* may be important in recruiting LAMs, lipids are considered to be the primary factor in inducing the LAM phenotype [19]. The underlying mechanisms for LAM induction are so far unknown.

In addition, the precise function of LAM in liver fibrosis remains obscure. Osteopontin, which is encoded by *SPP1* and highly expressed in liver LAMs, has previously been implicated in stimulating collagen I production from HSCs [81] and fibrogenesis in response to liver injury [82]. Blocking osteopontin in animal models of NASH demonstrates a protective effect [83–85]. However, transcriptional comparisons of liver LAMs derived from animals fed with standard diet and western diet (WD) highlight a more mature phenotype of LAMs from WD-fed animals, and with lower expression levels of *Il1b, Tnf,* and *Il10*, suggesting a possible protective effect of LAMs [19]. Functional study of LAMs in adipose tissues through a *Trem2* knockout animal suggests protective roles of LAMs in lipid uptake and thus preventing adipocyte hypertrophy [80]. However, *Trem2* knockout may also have different functional impacts other than LAM induction. Generation of clean “*LAM knockout*” animals seems to be necessary. In addition, differently from adipose LAMs, hepatic LAMs do not possess enhanced ability in lipid uptake as compared to resKCs [14], suggesting functional variabilities in LAMs from different tissues.

In summary, the discovery of LAMs by scRNA-seq technologies raises more enthusiasm to reevaluate the classification and the functions of liver macrophages during fibrogenesis. With the continuous discovery of LAM-like cells in multiple tissues, including the brain, the lung, and the adipose tissue as previously mentioned [80, 86, 87], functional contribution of LAMs to tissue homeostasis as well as the regulatory mechanisms under LAM induction and maintenance in different tissue contexts warrant future studies.

## Perspectives and conclusions

With the continuous development of multiple powerful technologies in the single-cell field, the scRNA-Seq technologies are transforming our understanding of liver biology and disease pathogenesis. The mostly widely adopted scRNA-seq techniques in current studies include 10× Chromium, Smart-Seq2, CEL-Seq2, and Drop-Seq. Pros and cons with each technique are discussed elsewhere in detail [88, 89]. Appropriate technique or combination of techniques should be chosen in the context of the study design and the study aims. For example, when studying rare cell types or aiming to identify lowly expressed genes or splicing variants, Smart-seq2 is preferred, as it has full-length gene coverage, whereas in CEL-Seq2 and 10× Chromium only the 3ʹ part of the gene is sequenced. However, the high cost of Smart-seq2 hindered its utility in high-throughput analysis for broader cell coverage, in which 10× Chromium is frequently adopted (Table 1). Although the scRNA-seq technology is powerful in tackling many biological questions, the low number of transcripts detected in each single cell makes it sometimes hard to fully represent cell diversity, presenting one major drawback of this technology. Enhanced gene coverage with technical improvement, or additional amplification step for genes-of-interest, may help partly solve this issue. In addition, many complementary techniques can facilitate to accurately define a subpopulation, including bulk RNA-seq, proteomics, fate-mapping, and mass cytometry.

Furthermore, new technologies aiming to integrate multimodal information of a single cell at different levels are evolving, such as the spatial scRNA-seq [90–92], single-cell epigenomics [93], single-cell genome sequencing [94], spatial proteomics [19, 95, 96], and spatial metabolomics [25]. These approaches will generate additional data on phenotypes (or even genotypes) of a given cell, enabling more comprehensive analyses of different cell types and cellular states under physiological and pathological conditions. The challenge now is to translate new knowledge into effective novel therapeutic treatments.

Several classes of pharmacological agents targeting liver fibrosis have been developed in targeting hepatocyte apoptosis, inflammation, myofibroblast activation, etc [3–6]. New knowledge obtained with scRNA-seq studies of liver fibrosis will help discover novel targets at new levels of precision. For example, the identification of LPAR1 with specific expression in collagen-producing HSC subsets allows the discovery of potential new strategies to target-activated HSCs [46]. Besides, although future functional validations are warranted, the discovery of the scar-associated endothelial subsets with potential immunomodulatory function [10], and the autocrine IL11 signaling that accounts for stimulated stellakine secretion from activated HSCs in liver fibrosis [12] may offer new targets to modulate immune responses specifically during liver fibrogenesis. In addition, the evolutionarily conserved ALK1-BMP9/10 axis between KCs and stellate cells is identified to be crucial in regulating KC maintenance in the liver, providing a potential target to prevent the loss of KCs in liver fibrosis [19]. The new subset of macrophages LAMs that was discovered to be closely associated with liver steatosis and fibrogenesis may also provide a potential target to modulate liver immune responses and fibrogenesis. ScRNA-seq studies of liver fibrosis also reveal exciting new discoveries of other immune cells besides macrophages, such as T cells, dendritic cells, etc [8]. These discoveries will also lead to new opportunities to target profibrogenic hepatic inflammation.

In conclusion, single-cell technologies have already yielded transformative new discoveries in liver biology and fibrogenesis at an unprecedented resolution. Further efforts are urged to fully understand the functional contributions of specific alterations in cell subsets or cellular status during liver fibrogenesis, and to translate these findings into effective therapeutic approaches.
